# Impact of gas background on XFEL single-particle imaging

**DOI:** 10.1038/s41598-025-15092-8

**Published:** 2025-08-12

**Authors:** Tong You, Johan Bielecki, Filipe R. N. C. Maia

**Affiliations:** 1 Laboratory of Molecular Biophysics, Institute for Cell and Molecular Biology, Uppsala University, Box 596, 75124 Uppsala, Sweden; 2 European XFEL, Holzkoppel 4, 22869 Schenefeld, Germany; 3 Lawrence Berkeley National Laboratory, NERSC, Berkeley, CA 94720 USA

**Keywords:** X-rays, Imaging techniques, Macromolecules and clusters

## Abstract

Single-particle imaging (SPI) using X-ray free-electron Lasers (XFELs) offers the potential to determine protein structures at high spatial and temporal resolutions without the need for crystallization or vitrification. However, the technique faces challenges due to weak diffraction signals from single proteins and significant background scattering from gases used for sample delivery. A recent observation of a diffraction pattern from an isolated GroEL protein complex Ekeberg T et al. (Light Sci Appl 13:15, 2024. 10.1038/274s41377-023-01352-7) had similar numbers of signal and background photons. Ongoing efforts aim to reduce the background created by sample delivery, with one approach replacing most of the used gas with helium Yenupuri T et al. (Sci Rep 14:4401, 2024. 10.1038/s41598-024-54605-9). In this study, we investigate the effects of a reduced background on the resolution limits for SPI of isolated proteins under different experiment conditions. As a test case, we used GroEL, and we used experimentally derived parameters for our simulations. We observe that background significantly impacts the achievable resolution, particularly when the signal strength is comparable to the background. This is best exemplified at 6.0 keV, where a background reduction by a factor of 10 leads to a resolution improvement from 1.9 to 1.2 nm, for a dataset of $10^4$ patterns.

## Introduction

Unlike traditional synchrotron X-ray sources, X-ray Free Electron Lasers (XFELs)^1,2^ provide orders-of-magnitude higher peak intensities and ultrashort pulse durations, typically on the femtosecond scale^3^. Using them it may be possible to image individual biological particles to high spatial and temporal resolution, using a technique known as single-particle imaging (SPI)^4–6^. This enables imaging ultrafast dynamics^7–9^ and offers advantages over X-ray crystallography and cryo-electron microscopy (cryo-EM). In particular, SPI circumvents the need to crystallize the sample, as required in X-ray crystallography, and in principle allows much higher time resolution than cryo-EM^10,11^.

The potential of SPI was first theorized by Neutze et al.^12^, who predicted that using sufficiently short XFEL pulses could allow proteins to diffract before the intense ionizing radiation destroys the sample. This “diffraction-before-destruction” concept was experimentally validated by Chapman et al. in 2006^13^, demonstrating the feasibility of obtaining structural information from objects before they are vaporized by the beam.

However, significant challenges remain for SPI to achieve high-resolution reconstructions of single proteins^14,15^. One of the primary difficulties arises from the weak diffraction signal generated by single proteins, as SPI does not benefit from the signal enhancement provided by the numerous sample copies that make up the crystal lattice in X-ray crystallography. Improvements in X-ray optics^16,17^, leading to increased fluence, present a possible solution, but at the cost of a smaller beam cross-section and decreased probability of intersection. Delivering the sample to the beam in its near-native conformation is another challenge. Various approaches, including gas dynamic virtual nozzles (GDVNs)^18,19^ and electrospray ionization (ESI)^20^, have been developed to introduce proteins into the XFEL beam. ESI, in particular, has been invaluable in delivering small samples, as its minute droplets are essential to minimize contaminants present in the sample solution, a common problem with GDVN-based sample delivery^21^. But the gas mixture used for ESI, a mixture of carbon dioxide and nitrogen, produces significant background photons, especially when compared to the signal from a single protein.

Despite advances in sample delivery, SPI has so far achieved only two-dimensional reconstructions of cells^22,23^ and three-dimensional reconstructions of viruses^24,25^. A complete three-dimensional reconstruction of a single protein remains elusive. Recently, the first diffraction pattern from a protein complex, GroEL, was recorded^26^. Although many patterns were collected, only one could confidently be attributed to a single GroEL protein complex, highlighting the difficulty of obtaining high-quality diffraction data from smaller particles.

Several factors contribute to the challenges of SPI with proteins. The smaller size of proteins inherently reduces the scattering signal compared to larger particles such as viruses. Additionally, background noise further complicates the reconstruction process. While there are numerous simulation studies of SPI^27–31^, only a few incorporate external noise sources. While some have focused on the impact of detector noise^32^, and other works have examined radiation damage^33–36^ and water layer effects^37,38^, background gas scattering has received little attention. Recent improvements in ESI, replacing most of the gas used with helium^39^, have significantly reduced sample delivery related background scattering, potentially improving the signal-to-noise ratio for protein diffraction^40^.

In this work, we investigate the effects of background scattering on the quality of reconstructed protein structures. An important difficulty when doing these types of numerical studies is simulating realistic signal strength while taking into account effects such as radiation damage and the fraction of the X-ray beam present in its focus, as calculating these from first principles is not trivial. To overcome this problem we introduce the concept of useful fluence. By useful fluence, we mean the fluence which would give rise to an observed pattern if the sample were immune to radiation damage. This is always smaller than the actual fluence as the scattering cross-section is reduced due to radiation damage, especially at soft X-ray energies. A key advantage of useful fluence is that it can be derived from experimental data of known samples. In this way, the difficulties mentioned above are implicitly taken into account as the experimental data already includes those effects, i.e. real radiation damage and focusing optics. Furthermore we incorporated experimentally measured gas scattering, aiming to provide a realistic assessment of the achievable resolution in SPI experiments. Our results show that background scattering significantly influences the resolution of the reconstructions as well as the number of diffraction patterns required and hope these can help guide future experimental designs.

## Results and discussion

We chose the *Escherichia coli* chaperonin GroEL^41^ to look at the effects of experimental background on the resolution limits because it was the first isolated protein observed at an XFEL^26^, and background noise was a limiting factor in that experiment. GroEL has also been extensively characterized in past studies, and many high-resolution X-ray structures are available.

**Fig. 1:**
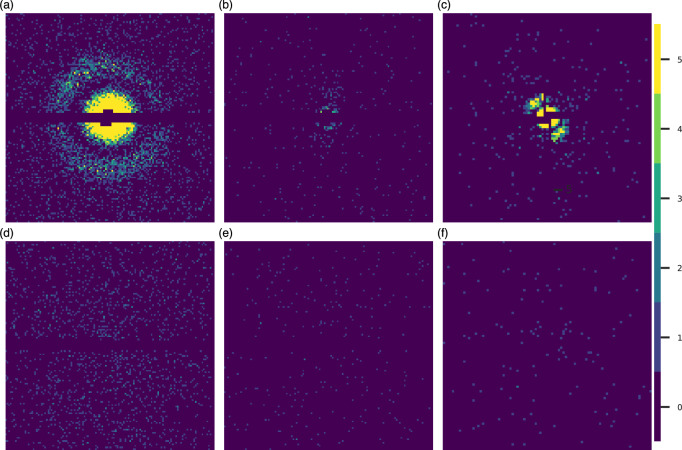
Diffraction patterns under medium noise conditions. The top row shows protein diffraction combined with background at (**a**) 1.2 keV, (**b**) 2.5 keV, and (**c**) 6.0 keV. The bottom row shows background only scattering for (**d**) 1.2 keV, (**e**) 2.5 keV, and (**f**) 6.0 keV. The color bar denotes photon counts per pixel.

We simulated diffraction patterns of randomly oriented single GroEL particles, from the PDB model 1SS8^42^, using experimentally measured parameters for the Small Quantum Systems (SQS)^43^ and the Single Particles, Clusters, and Biomolecules & Serial Femtosecond Crystallography (SPB/SFX)^44^ instruments of the European XFEL at 1.2, 2.5 and 6.0 keV (see 2D pattern simulation in the Methods section for exact details).

We combined the simulated (noiseless) patterns with experimentally measured background^26^, obtained at 1.2 keV, by incoherent addition. The background for the 2.5 keV simulations was the same as the 1.2 keV, rescaled to take into account the reduction in pulse energy at 2.5 keV, compared to 1.2 keV at the SQS beamline, which we estimated at $3\times$ less. As experimental background was not available at 6.0 keV we modeled it based on the low energy (1.2 keV) background (see Background modeling in Methods). After the incoherent addition we Poisson-sampled the result to obtain the number of photons per pixel (see Fig. 1 for examples).

Given recent developments in sample delivery injection^40^, the background in future experiments is expected to be significantly reduced. To investigate the consequences of this, we did simulations with $10\times$ and $100\times$ less background. The three background levels will be referred to as high, medium, and low background, respectively. We also performed Poisson sampling on just the noiseless simulations to establish a baseline, which we refer to as zero background.

The number of recorded diffraction patterns is also an important parameter since averaging techniques can boost the signal-to-noise ratio. We tested datasets with $10^3$, $10^4$, and $10^5$ patterns.

**Table 1:** Average photon counts for the signal expected from a single GroEL particle along with the background for each level investigated.

	1.2 keV	2.5 keV	6.0 keV
GroEL signal	5644	101	1048
High background	13967	2235	1044
Medium background	1397	223	104
Low background	140	22	10

Since signal-to-noise is an important aspect of the present study, Table 1 shows the average number of photons for a single GroEL particle along with the background at the three investigated levels .

To orient the 2D patterns into a consistent 3D volume we used the Dragonfly^45^ package which employs the Expand-Maximize-Compress (EMC) algorithm^46^. In principle one can perform background correction either before or after the EMC assembly. We found that correcting the 2D diffraction patterns leads to instabilities in the EMC assembly and failure to converge. Instead, we did two separate EMC assemblies, one of the background-corrupted protein diffraction signal and another one of only the background diffraction which we subtracted from the former.

**Fig. 2:**
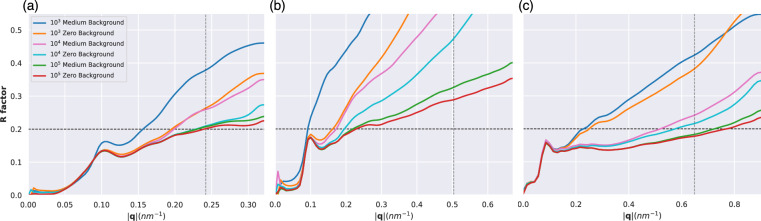
R factor curves for all three geometries under medium and zero background conditions for (**a**) 1.2 keV, (**b**) 2.5 keV and (**c**) 6.0 keV. The horizontal dashed line corresponds to the 0.2 threshold used to define the resolution.

To monitor the quality of the assembled 3D intensities we used the R-factor^47^, between the assembled intensities and the scattering factors calculated from the PDB model. We define R-factor resolution as the resolution where the R factor curve intersects the 0.2 threshold. For all three energies, the R-factor resolution improves as the number of patterns increases, or when noise decreases (see Table 2).

It was recently shown to be possible to achieve background levels similar to our medium background case^40^, so that is where we will focus the discussion. Fig. 2 shows the zero and medium background R-factor curves for all datasets. The background impact on the EMC intensity assembly is largest when only $10^3$ patterns are used. This is unsurprising as the signal in a dataset scales with the number of images, but the background only scales as the square root of the number of images as the mean of the background can be subtracted. This is also apparent from the speckle visibility of slices through the assembled volume, as seen in Figs. S9, S10, and S11. The R factor curves under high and low noise conditions can be found in Figs. S1 and S4.

The recovered 3D intensity volumes were then phased with libspimage^48^ (see methods for more details). We estimated the quality of the reconstructed structures using the Phase Retrieval Transfer Function (PRTF)^49^ and the Fourier Shell Correlation^50^ (FSC).

**Fig. 3:**
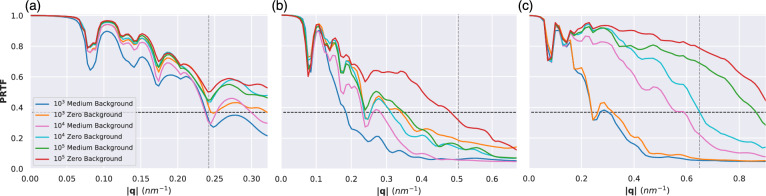
PRTF curves for all three geometries under medium and zero background conditions for (**a**) 1.2 keV, (**b**) 2.5 keV and (**c**) 6.0 keV. The horizontal dashed line corresponds to the 1/e threshold traditionally used to define the resolution. The vertical dashed line indicates the edge resolution for each geometry.

The PRTF curves for the medium noise datasets are shown in Fig. 3. For all three photon energies phasing is more reproducible (high PRTF) for larger datasets. This can also be seen from the resolution determined from the PRTF in Table 2. This is also the case for the high- and low-noise datasets. The PRTF improvement with dataset size is more apparent for the 6.0 keV datasets (Fig. 3(c)). The PRTF curves for $10^3$ patterns with and without background have the same resolution (3.3 nm and 3.1 nm), whereas the resolution for both the $10^4$ and $10^5$ dataset reconstructions is twice (1.7 nm and 1.5 nm) and nearly thrice (1.2 nm and 1.3 nm) as good, respectively. This shows how the resolution is limited by the number of patterns for the 6.0 keV reconstructions. PRTF curves for the high and low noise datasets can be found in Figs. S2 and S5.

**Table 2:** Comparison of multiple resolution metrics for $10^3$, $10^4$, and $10^5$ datasets. For each condition, the R-factor resolution is given, followed by the PRTF and finally the FSC, the three values separated by forward slashes. For a precise description of how resolutions are calculated, refer to the Methods section.

Resolutions in nm					
Photon energy (keV)	Dataset size	High	Medium	Low	Zero background
1.2	10^3^	6.9 / 4.7 / 3.5	6.4 / 3.9 / 3.2	5.2 / 3.1 / 3.1	5.1 / 3.1 / 3.2
1.2	10^4^	5.5 / 4.2 / 3.3	5.0 / 3.3 / 3.1	4.4 / 3.1 / 3.1	4.4 / 3.1 / 3.1
1.2	10^5^	5.2 / 4.1 / 3.2	4.3 / 3.1 / 3.1	4.2 / 3.1 / 3.1	4.2 / 3.1 / 3.1
2.5	10^3^	–	10.9 / 5.3 / 6.2	11.1 / 5.0 / 4.8	6.2 / 3.0 / 2.8
2.5	10^4^	–	5.8 / 3.8 / 2.8	5.3 / 3.4 / 2.8	5.1 / 3.1 / 2.7
2.5	10^5^	6.7 / 4.4 / 2.9	4.6 / 2.8 / 2.3	4.7 / 2.0 / 1.6	4.4 / 2.0 / 1.5
6.0	10^3^	5.1 / 4.2 / 2.9	4.4 / 3.3 / 2.7	4.2 / 3.1 / 2.6	4.0 / 3.0 / 2.6
6.0	10^4^	4.0 / 2.4 / 1.9	1.9 / 1.7 / 1.2	1.8 / 1.6 / 1.1	1.7 / 1.5 / 1.1
6.0	10^5^	1.7 / 1.4 / 1.1	1.4 / 1.2 / 1.1	1.3 / 1.1 / 1.1	1.3 / 1.1 / 1.1

The FSC is the most common method to assess the resolution of a reconstruction in cryo-EM. Since there is a ground-truth structure available, there is no requirement to separate the experimental data into two separate half-data sets and instead we can compare directly to the ground-truth (see Methods on details). The resolution determined from the FSC, using the half-bit curve criterion^51^, was always equal to or better than the one determined from the PRTF.

**Fig. 4:**
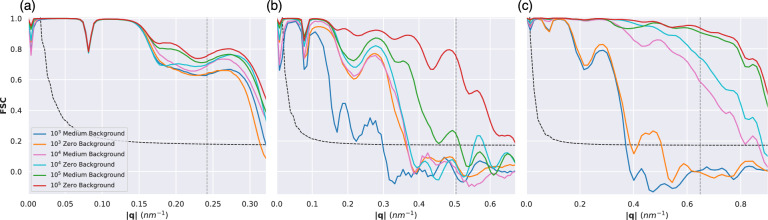
Radially averaged FSC curves under medium and zero background conditions of electron density reconstructions for **(a)** 1.2 keV, **(b)** 2.5 keV and **(c)** 6.0 keV. The vertical dashed gray line represents the edge of the detector. The half-bit curve is shown as a black dashed line.

FSC curves for the medium noise reconstructions are shown in Fig. 4 (see Figs. S3 and S6 for high and low noise FSC curves). The biggest resolution gains can be observed at 6.0 keV when the number of patterns is increased from $10^3$ to $10^4$ at each noise level (a 50% increase for high noise, 125% for medium noise, and 135% for low noise). The number of patterns seems to make the most difference for the 6.0 keV dataset, where it is clear that the correlation stays higher over a larger q-range when the number of patterns is increased. There is a clear separation between the FSC curves for $10^3$ and $10^4$ patterns. At the same noise level, more gains can be had by going from $10^3$ to $10^4$ patterns (an improvement of more than a factor of 2 in the resolution), compared to going from $10^4$ to $10^5$ patterns (almost no improvement). These improvements can not be seen for the 1.2 keV reconstructions. The only other clear improvement that can be seen going from $10^3$ to $10^4$ patterns is for the medium noise 2.5 keV reconstructions.

**Fig. 5:**
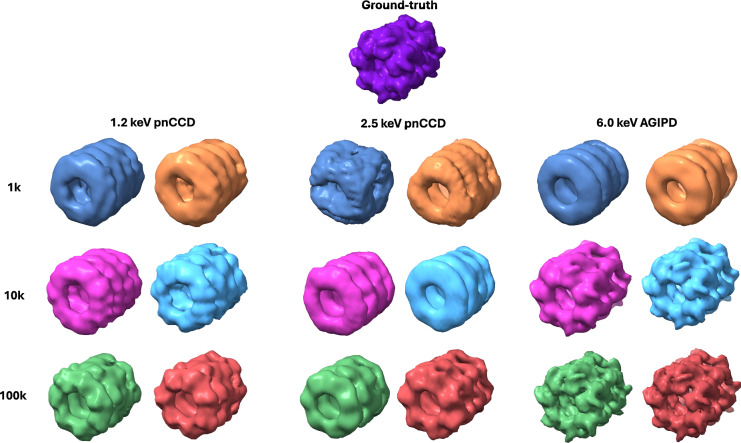
Reconstructed electron density for all three energies for $10^3$, $10^4$, and $10^5$ pattern reconstructions. For each energy, the left column (in blue, magenta, and green) represents the medium background condition, and the right column (in orange, cyan, and red) represents the zero background condition. The ground truth is shown on the top row.

It is also important to look at the reconstructions visually to gain an intuitive understanding of the effect of the background-induced resolution degradation on the electron density map (see Fig. 5). For the reconstructions under high and low noise conditions, see Figs. S7 and S8. We can see that the differences between the medium background and the zero background reconstructions reduce significantly as the dataset sizes increase, in line with our previous findings. For small dataset sizes, such as $10^3$ patterns at 2.5 keV, the noise can make the GroEL iconic barrel structure unrecognizable.

**Fig. 6:**
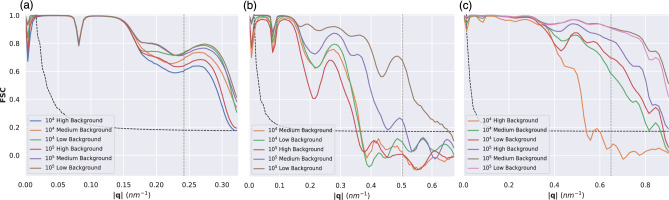
FSC curves under high, medium, and low noise conditions for $10^4$ and $10^5$ datasets for (**a**) 1.2 keV, (**b**) 2.5 keV and (**c**) 6.0 keV. The vertical dashed gray line represents the edge of the detector. The curved dashed black line marks the half-bit curve. The resolution, as determined by the FSC, is mainly limited by the experimental geometry for 1.2 keV. At 2.5 and 6.0 keV both the noise and the number of patterns limit the resolution, although the resolution improvements are larger at 6.0 keV.

Both a decrease in the background noise or an increase in the number of diffraction patterns used in a reconstructions lead to improved resolutions. To investigate the relative contribution of these two factors (so we can decide whether to focus on noise reduction or larger number of images), we compare the FSC curves at different noise and dataset sizes in Fig. 6.

For 1.2 keV there is very limited improvement in the FSC, regardless of background or dataset size. Here, the main limitation is the experimental geometry, as even for high background and $10^4$ images the FSC is comfortably above the half-bit threshold at the edge of the detector, marked by the gray dashed vertical line. For 2.5 keV, both a background reduction and an increase in dataset size led to better resolutions. In certain cases a $10\times$ increase in background can be compensated by a $10\times$ increase in dataset size, as seen in the cases of $10^5$ high background and $10^4$ medium background which give similar curves. For $10^4$ images a decrease in background from medium to low does not led to better resolution. But at $10^5$ images the same background decrease result in a significant improvement in the FSC. This points that increases in dataset size must be done in parallel with decreases in background noise, or we quickly hit diminishing returns. For 6.0 keV we observed the largest improvements in resolution as a function dataset size or going from high to medium background. Decreasing the background from medium to low has little impact, as the medium background is already substantially weaker than the signal. And an order of magnitude increase in dataset size is superior to a decrease in background by two orders of magnitude, i.e. comparing high background $10^5$ images to low background $10^4$ images. Just as for 1.2 keV we are also limited by the experimental geometry for several conditions, showing that there is potential for further improvements in resolution, as long as the required dataset sizes can be achieved.

## Methods

### 2D pattern simulation

Diffraction patterns were simulated with Condor^52^ with the parameters shown in Table 3. The orientation of each particle was randomized, and the horizontal polarization of the EuXFEL beam was taken into account.

**Table 3:** Key simulation parameters for each geometry investigated. The photon useful fluence scale covers three orders-of-magnitude. The detector parameters for the 1.2 and 2.5 keV correspond to a pnCCD^53^, one of the detectors available at SQS, binned $8 \times 8$. For 6.0 keV they correspond to an AGIPD^54^, the main detector at SQB/SFX, cropped to a square and binned $12 \times 12$. Downsampling was done to reduce computational time while maintaining sufficient oversampling.

	1.2 keV	2.5 keV	6.0 keV
Edge resolution (nm)	4.13	1.98	1.51
Corner resolution (nm)	2.99	1.43	1.07
Pixel size (µm)	75	75	200
Number of pixels	1024 × 1024	1024 × 1024	1256 × 1092
Downsampled pixels	128 × 128	128 × 128	91 × 91
Oversampling	17.2	8.27	4.59
Useful Fluence (photons/µm^2^)	1.46 × 10^12^	2.33 × 10^11^	1.04 × 10^13^
Useful Fluence (µJ/µm^2^)	280	93	10000

A 1024 × 1024 pixels pnCCD detector^53^ with 75 µm pixel size and 0.15 m distance to the interaction point was simulated with photon energies of 1.2 and 2.5 keV. Since successful phase retrieval only requires oversampling larger than 2, the computational load was reduced by downsampling the detector to 128 by 128 pixels. The 1.2 (2.5) keV simulations have an edge resolution of 4.13 (1.98) nm and a maximum corner resolution of 2.99 (1.43) nm.

To simulate the Adaptive Gain Integrating Pixel Detector (AGIPD)^54^, the EXtra-geom module^55^ from the European XFEL was used to assemble the 16 detector modules into a single image. The default geometry in EXtra-geom was used, resulting in an image with 1256 × 1092 pixels. The AGIPD detector has a pixel size of 200 µm. The whole AGIPD detector was first cropped to a square of size 1092 × 1092, then the detector was downsampled to a size of 91 × 91 pixels using a 12 × 12 window. This downsampled detector has an edge resolution of 1.51 nm and a corner resolution of 1.07 nm.

Our simulations do not directly model radiation damage, as we use atomic form factors from the neutral atoms. Instead, we reduced the incident fluence to the useful fluence, that is, the fluence which would give rise to an observed diffraction pattern, if the sample were immune to radiation damage, and which can be experimentally estimated using samples of known size and composition^56^. For 1.2 keV we used a useful fluence of 280 µJ/µm^2^, to match a recent experiment^26^. For 2.5 keV, we did not have experimental data, so we estimated a useful fluence of 93 µJ/µm^2^, a 3× reduction resulting simply from the difference in typical pulse energies observed at these two photon energies at the SQS beamline, likely an underestimate. For 6.0 keV, we used a useful fluence of 10 mJ/µm^2^ in line with experiments^57^ using the nano-KB mirrors^58^.

### Background modeling

In an SPI experiment using an aerodynamic lens-stack, the background from elastic scattering on gas consists of two components. The first component, $I_{\textrm{line}}$ results from the scattering of gas at relatively uniform pressure *p* extending from the interaction point to the detector. The second contribution, $I_{\textrm{jet}}$ comes from the scattering of the higher-pressure gas jet immediately under the outlet of the aerodynamic lens.

We describe the rotationally averaged scattering arising from a gas molecule by the Debye scattering formula, using $q=2 \sin {\theta }/\lambda$, where $\theta$ is half the scattering angle.

1 $$\begin{aligned} I(q) = {{\sum }}_i {{\sum }}_j f_i f^*_j \frac{\sin (2 \pi q d_{ij})}{2 \pi q d_{ij}}, \end{aligned}$$

where *i*, *j* goes over all atoms in the molecule, $f_i$ is the atomic form factor for atom *i*, and $d_{ij}$ is the distance between atom *i* and *j*. The range of scattering vectors covered by the detector varies as a function of the longitudinal distance *l* between the gas molecule and detector plane. For a detector pixel with physical coordinates (*x*, *y*), we have $\theta = \frac{\arctan {(r/l)}}{2}$ where $r=\sqrt{x^2+y^2}$. Using this explicit expression for $\theta$ we define $I(r,l,\lambda )$ as the scattering from a gas molecule at a distance *l* from the detector towards a pixel located at radius *r*.

Hence,

2 $$\begin{aligned} I_{\textrm{line}}(r,\lambda )=N_{ph}n r_0^2\Omega \int _0^l I(r,l,\lambda )dl \end{aligned}$$

where $\Omega$ is the solid angle of the pixel, $r_0$ is the classical electron radius, *n* is the number of gas molecules per $\text {m}^3$, known as the volumetric number density, and $N_{ph}$ is the number of photons in the XFEL pulse.

Analogously we model $I_{jet}$ as:

3 $$\begin{aligned} I_\textrm{jet}(r,\lambda )=N_{ph}n_{jet} r_0^2\Omega \int _{l-\Delta l/2}^{l+\Delta l/2} I(r,l,\lambda )dl \end{aligned}$$

where $\Delta l$ is the gas jet width and $n_{jet}$ its volumetric number density.

The volumetric number density of background gas can be well approximated by the ideal gas law, $n=p/kT$, while $n_{jet}$ is related to the flux of gas molecules through the aerodynamic lens. The total gas background can be expressed as $I_{gas}=I_{line} + I_{jet}$, with $n_{jet} = c \cdot n$, where *c* equals the relative pressure increase in the gas jet relative the overall chamber pressure. $\Delta l$ is typically not known. However, the *q* dependence of $I_{\textrm{jet}}$ is independent of $\Delta l$ as long as $\Delta l \ll l$. It is thus possible to assume a small $\Delta l$ value and fit *c* to a measured gas background. As *c* only depends on the gas flow out of the aerodynamic lens, one such fit is sufficient to determine the gas background for different $\lambda$ and *l* values. Here we use a gas background measured at 1.2 keV, and obtain $c=1950$.

### 3D intensity assembly

Dragonfly^45^ was used to assemble the diffraction patterns. Rotational sampling was optimized to ensure that the average number of patterns per orientation was close to one. For $10^3$, $10^4$, and $10^5$ patterns, n = 3 (1380), n = 6 (10680), and n = 12 (86520) rotational samples were used, respectively. In cases where high background noise relative to the protein signal led to convergence issues (i.e., pattern collapse into a few orientations), the rotational sampling was reduced to prevent this collapse and ensure proper reconstruction. Fluence scaling was enabled for all EMC reconstructions, as during an experiment the fluence on the sample is unknown. The recovered fluence was almost identical for all patterns, as expected. Due to potential instabilities in high signal, high background, high rotational sampling, or low pattern reconstructions, the deterministic annealing variant of EMC was utilized, with the annealing parameter $\beta$. This $\beta$ was gradually raised to 1.0 over the assembly. For high signal datasets (the $10^5$ with background and zero background) the initial $\beta$ was 0.001. For all the other reconstructions it was 0.01. The assembly was monitored until the mutual information and average log-likelihood plateaued at which point the iterations were stopped. The total number of iterations ranged from 180 to 650.

### 3D phase retrieval

Three-dimensional phase reconstructions were performed using libspimage^48^. Each reconstruction consisted of 500 Relaxed Averaging Alternating Reflections (RAAR)^59^ iterations followed by 450 Error Reduction (ER)^60^iterations. The RAAR feedback parameter $\beta$ was adjusted based on noise level: for 6.0 keV data, $\beta$ was set from 0.80 at the start to 0.85 at the end of reconstruction for all of the background-corrupted reconstructions and the $10^3$ zero background reconstruction and 0.90 at the start to 0.95 at the end of the reconstruction for the $10^4$ and $10^5$ the zero background reconstructions. For 2.5 keV at high and medium background and $10^3$ low background it went from 0.60 to 0.65 and for all other reconstruction it went from 0.70 to 0.75. The support was updated every 20 iterations using a volume-constraining version of the shrinkwrap algorithm^61^ (where the total support volume is enforced at every support update iteration) with Gaussian blurring with standard deviations decreasing from 1.5 to 1.0 voxels. The initial estimate for the volume of the support was 2380 $\text {nm}^3$ and this was reduced to 2190 $\text {nm}^3$ at the end of the reconstruction.

500 independent reconstructions were performed for each dataset, and the 450 with the lowest real-space error were selected for alignment and averaging. The Phase Retrieval Transfer Function (PRTF)^49^ was used to assess reproducibility and determine spatial resolution by calculating the intersection of the radial averaged of the critically sampled PRTF^25^ (the oversampled PRTF convoluted with the Fourier transform the convex hull of the support) with the 1/*e* threshold. Reconstructions were performed with the negative voxels in Fourier space free to take any intensity value.

### 3D structure alignment

To calculate the FSC between our reconstructions and the ground truth, the 1SS8 PDB model, we have to rotationally and translationally aligned them. This was done using an automated script in ChimeraX^62^. An electron density at a resolution of 15 Å was generated from the PDB using the molmap command in ChimeraX. Both the PDB-generated electron density and reconstructed electron densities were centered. Then 30 iterations of translational alignment were performed, followed by 30 iterations of rotational alignment. After the structures were aligned, the FSC was calculated by radially averaging the 3D correlation map using 1 voxel thick spherical shells. The resolution was determined by calculating the first intersection of the FSC with the (modified) half-bit threshold curve^51^. The above procedure was repeated for the centrosymmetric version of each reconstruction, due to the handedness ambiguity inherent in all phasing procedures, and the one with the best FSC was kept.

### 3D EMC model alignment

3D scattering intensities were generated from the PDB model of GroEL using Condor^52^. The EMC assembled intensities were aligned to this ideal model intensities using ChimeraX and R-factors were calculated. We calculate the R factor until and including a given resolution^47^. To determine the R-factor resolution, we find the first intersection with a constant threshold of 0.2.

## Conclusion

In this study, we demonstrated that background noise significantly influences the achievable resolution in our imaging system. Using a 6 keV nano-focus beam and collecting $10^5$ patterns, we achieved the best resolution of approximately 1.1 nm. While this is insufficient for direct atomic modeling it could still give important information about large-scale changes in macromolecular systems. For example, by studying large conformational changes, or binding, in a time-resolved manner particularly when the static structure of the sample is known. However, collecting large datasets using the nano-focus beam, $\sim$200 nm diameter^58^, presents challenges compared to the micro-focus beam, $\sim$1.5 µm diameter^63^, as the smaller beam area reduces the interaction probability and the number of particles that can be imaged by $\sim$50$\times$. In this sense, comparing datasets of the same size for different focal spots does not take the inevitable time constraints of beamtimes into account. But this dataset size constraint also highlights the importance of background reduction, as it was possible to achieve the same resolution of 1.1 nm with only $10^4$ background-reduced patterns. Even for soft X-rays, a reduction in background significantly improves the resolution and, for example in the case of the smaller $10^3$ datasets at 2.5 keV, can be the difference between a failed and a successful reconstruction.

Looking forward, achieving sub-nanometer resolution for biological samples will require further technical advances, particularly in reducing the size of the X-ray focal spots, increasing fluence, and improving sample delivery precision to fully utilize the smaller beam sizes. Background reduction will also help improve the maximum attainable resolution. Pulses with higher peak power and shorter duration^64^ will reduce radiation damage, increasing the useful fluence. Another possibility is to enhance the diffraction signal through the use of transient resonances. A recent experiment showed a significant non-linear enhancement of the diffraction signal in Xenon clusters^65^. If those results can be extended to biological samples, then it would lead to an even greater improvement in the resolution that can be achieved.

## Supplementary Information

Supplementary Information.

## Data Availability

Data are available from the corresponding author upon reasonable request.

## References

[1] Ekeberg, T. et al. Observation of a single protein by ultrafast X-ray diffraction. *Light Sci. Appl.* **13**, 15. 10.1038/s41377-023-01352-7 (2024). 38216563 10.1038/s41377-023-01352-7 PMC10786860

[2] Yenupuri, T. et al. Helium-electrospray improves sample delivery in X-ray single-particle imaging experiments. *Sci. Rep.* **14**, 4401. 10.1038/s41598-024-54605-9 (2024). 38388562 10.1038/s41598-024-54605-9 PMC10883998

[3] Emma, P. et al. First lasing and operation of an ångstrom-wavelength free-electron laser. *Nat. Photonics* **4**, 641–647. 10.1038/nphoton.2010.176 (2010).

[4] Kang, H. et al. Hard X-ray free-electron laser with femtosecond-scale timing jitter. *Nat. Photonics* **11**, 708–713. 10.1038/s41566-017-0029-8 (2017).

[5] Spence, J. XFELs for structure and dynamics in biology. *IUCrJ* **4**, 322–339. 10.1107/S2052252517005760 (2017). 28875020 10.1107/S2052252517005760 PMC5571796

[6] McNeil, B. & Thompson, N. X-ray free-electron lasers. *Nature Photon* **4**, 814–821. 10.1038/nphoton.2010.239 (2010).

[7] Bielecki, J., Maia, F. R. N. C. & Mancuso, A. P. Perspectives on single particle imaging with x rays at the advent of high repetition rate x-ray free electron laser sources. *Struct. Dyn.* **7**, 040901. 10.1063/4.0000024 (2020). 32818147 10.1063/4.0000024 PMC7413746

[8] Sobolev, E. et al. Megahertz single-particle imaging at the European XFEL. *Commun. Phys.* **3**, 97. 10.1038/s42005-020-0362-y (2020).

[9] Young, L. et al. Roadmap of ultrafast x-ray atomic and molecular physics. *J. Phys. B: At. Mol. Opt. Phys.* **51**, 032003. 10.1088/1361-6455/aa9735 (2018).

[10] Gaffney, K. J. & Chapman, H. N. Imaging atomic structure and dynamics with ultrafast x-ray scattering. *Science* **316**, 1444–1448. 10.1126/science.1135923 (2007). 17556577 10.1126/science.1135923

[11] Nass, K. et al. Structural dynamics in proteins induced by and probed with X-ray free-electron laser pulses. *Nat. Commun.* **11**, 1814. 10.1038/s41467-020-15610-4 (2020). 32286284 10.1038/s41467-020-15610-4 PMC7156470

[12] Amann, S. J., Keihsler, D., Bodrug, T., Brown, N. G. & Haselbach, D. Frozen in time: analyzing molecular dynamics with time-resolved cryo-EM. *Structure* **31**, 4–19. 10.1016/j.str.2022.11.014 (2023). 36584678 10.1016/j.str.2022.11.014 PMC9825670

[13] Banari, A. et al. Advancing time-resolved structural biology: Latest strategies in cryo-EM and x-ray crystallography. *Nat. Methods*10.1038/s41592-025-02659-6 (2025). 40312512 10.1038/s41592-025-02659-6

[14] Neutze, R. et al. Potential for biomolecular imaging with femtosecond X-ray pulses. *Nature* **406**, 752–757. 10.1038/35021099 (2000). 10963603 10.1038/35021099

[15] Chapman, H. et al. Femtosecond diffractive imaging with a soft-X-ray free-electron laser. *Nature Phys.* **2**, 839–843. 10.1038/nphys461 (2006).

[16] Aquila, A. et al. The linac coherent light source single particle imaging road map. *Struct. Dyn. (Melville, N.Y.)* **2**, 041701. 10.1063/1.4918726 (2015). 10.1063/1.4918726 PMC4711616 26798801

[17] Bielecki, J., Maia, F. R. N. C. & Mancuso, A. P. Perspectives on single particle imaging with x rays at the advent of high repetition rate x-ray free electron laser sources. *Struct. Dyn. (Melville, N.Y.)* **7**, 040901. 10.1063/4.0000024 (2020). 10.1063/4.0000024 PMC7413746 32818147

[18] Yamada, J. et al. Extreme focusing of hard X-ray free-electron laser pulses enables 7 nm focus width and W intensity. *Nat. Photonics* **18**, 685–690. 10.1038/s41566-024-01411-4 (2024).

[19] Inoue, I. et al. Nanofocused attosecond hard x-ray free-electron laser with intensity exceeding . *Optica* **12**, 309–310. 10.1364/OPTICA.554954 (2025).

[20] DePonte, D. P. et al. Gas dynamic virtual nozzle for generation of microscopic droplet streams. *J. Phys. D Appl. Phys.* **41**, 195505. 10.1088/0022-3727/41/19/195505 (2008).

[21] Knoška, J. et al. Ultracompact 3D microfluidics for time-resolved structural biology. *Nat. Commmun.* **11**, 657. 10.1038/s41467-020-14434-6 (2029). 10.1038/s41467-020-14434-6 PMC6994545 32005876

[22] Yamashita, M. & Fenn, J. B. Electrospray ion source. Another variation on the free-jet theme. *J. Phys. Chem.* **88**, 4451–4459. 10.1021/j150664a002 (1984).

[23] Bielecki, J. et al. Electrospray sample injection for single-particle imaging with x-ray lasers. *Sci. Adv.* **5**, eaav8801. 10.1126/sciadv.aav8801 (2019). 31058226 10.1126/sciadv.aav8801 PMC6499549

[24] Hantke, M. et al. High-throughput imaging of heterogeneous cell organelles with an X-ray laser. *Nature Photon* **8**, 943–949. 10.1038/nphoton.2014.270 (2014).

[25] van der Schot, G. et al. Imaging single cells in a beam of live cyanobacteria with an X-ray laser. *Nat. Commun.* **6**, 5704. 10.1038/ncomms6704 (2015). 25669616 10.1038/ncomms6704

[26] Ekeberg, T. et al. Three-dimensional reconstruction of the giant mimivirus particle with an x-ray free-electron laser. *Phys. Rev. Lett.* **114**, 098102. 10.1103/PhysRevLett.114.098102 (2015). 25793853 10.1103/PhysRevLett.114.098102

[27] Lundholm, I. V. et al. Considerations for three-dimensional image reconstruction from experimental data in coherent diffractive imaging. *IUCrJ* **5**, 531–541. 10.1107/S2052252518010047 (2018). 30224956 10.1107/S2052252518010047 PMC6126651

[28] Poudyal, I., Schmidt, M. & Schwander, P. Single-particle imaging by x-ray free-electron lasers-how many snapshots are needed?. *Struct. Dyn. (Melville, N.Y.)* **7**, 024102. 10.1063/1.5144516 (2020). 10.1063/1.5144516 PMC7088463 32232074

[29] Nakano, M., Miyashita, O., Jonic, S., Tokuhisa, A. & Tama, F. Single-particle XFEL 3D reconstruction of ribosome-size particles based on Fourier slice matching: requirements to reach subnanometer resolution. *J. Synchrotron Radiat.* **25**, 1010–1021. 10.1107/S1600577518005568 (2018). 29979162 10.1107/S1600577518005568

[30] Nakano, M., Miyashita, O. & Tama, F. Molecular size dependence on achievable resolution from XFEL single-particle 3D reconstruction. *Struct. Dyn.* **10**, 024101. 10.1063/4.0000175 (2023). 36942031 10.1063/4.0000175 PMC10024609

[31] Tegze, M. & Bortel, G. Comparison of EMC and CM methods for orienting diffraction images in single-particle imaging experiments. *IUCrJ* **8**, 980–991. 10.1107/S205225252100868X (2021). 34804550 10.1107/S205225252100868X PMC8562656

[32] Donatelli, J. J., Sethian, J. A. & Zwart, P. H. Reconstruction from limited single-particle diffraction data via simultaneous determination of state, orientation, intensity, and phase. *Proc. Natl. Acad. Sci. U.S.A.* **114**, 7222–7227. 10.1073/pnas.1708217114 (2017). 28652365 10.1073/pnas.1708217114 PMC5514772

[33] Kim, Y. et al. Expected resolution limits of x-ray free-electron laser single-particle imaging for reamlistic source and detector properties. *Struct. Dyn.* **9**, 064101. 10.1063/4.0000169 (2022). 36411869 10.1063/4.0000169 PMC9675053

[34] Yoon, C. H. et al. A comprehensive simulation framework for imaging single particles and biomolecules at the european x-ray free-electron laser. *Sci. Rep.* **6**, 24791. 10.1038/srep24791 (2016). 27109208 10.1038/srep24791 PMC4842992

[35] Fortmann-Grote, C. et al. Start-to-end simulation of single-particle imaging using ultra-short pulses at the European X-ray Free-Electron Laser. *IUCrJ* **4**, 560–568. 10.1107/S2052252517009496 (2017). 28989713 10.1107/S2052252517009496 PMC5619849

[36] Östlin, C., Timneanu, N., Caleman, C. & Martin, A. V. Is radiation damage the limiting factor in high-resolution single particle imaging with x-ray free-electron lasers?. *Struct. Dyn. (Melville, N.Y.)* **6**, 044103. 10.1063/1.5098309 (2019). 10.1063/1.5098309 PMC6701976 31463335

[37] Stransky, M. et al. Computational study of diffraction image formation from XFEL irradiated single ribosome molecule. *Sci. Rep.* **14**, 10617 (2024). 38720133 10.1038/s41598-024-61314-w PMC11078940

[38] Stransky, M. et al. Effects of radiation damage and inelastic scattering on single-particle imaging of hydrated proteins with an X-ray Free-Electron Laser. *Sci. Rep.* **11**, 17976. 10.1038/s41598-021-97142-5 (2021). 34504156 10.1038/s41598-021-97142-5 PMC8429720

[39] Stransky, M. et al. Water layer and radiation damage effects on the orientation recovery of proteins in single-particle imaging at an X-ray free-electron laser. *Sci. Rep.* **13**, 16359. 10.1038/s41598-023-43298-1 (2023). 37773512 10.1038/s41598-023-43298-1 PMC10541445

[40] Rafie-Zinedine, S. et al. Enhancing electrospray ionization efficiency for particle transmission through an aerodynamic lens stack. *J. Synchrotron Radiat.* **31**, 222–232. 10.1107/S1600577524000158 (2024). 38306300 10.1107/S1600577524000158 PMC10914161

[41] Hayer-Hartl, M., Bracher, A. & Hartl, F. U. The GroEL-GroES chaperonin machine: A nano-cage for protein folding. *Trends Biochem. Sci.* **41**, 62–76. 10.1016/j.tibs.2015.07.009 (2016). 26422689 10.1016/j.tibs.2015.07.009

[42] Chaudhry, C., Horwich, A. L., Brunger, A. T. & Adams, P. D. Exploring the Structural Dynamics of the E.coli Chaperonin GroEL Using Translation-libration-screw Crystallographic Refinement of Intermediate States. *J. Mol. Biol.* **342**, 229–245. 10.1016/j.jmb.2004.07.015 (2004). 15313620 10.1016/j.jmb.2004.07.015

[43] Meyer, M. et al. The Small Quantum System (SQS) Instrument at European XFEL: Results of commissioning and first experiments, vol. 1412 (IOP Publishing, 2020) 112005.

[44] Mancuso, A. P. et al. The single particles, clusters and biomolecules and serial femtosecond crystallography instrument of the european XFEL: Initial installation. *J. Synchrotron Radiat.* **26**, 660–676. 10.1107/S1600577519003308 (2019). 31074429 10.1107/S1600577519003308 PMC6510195

[45] Ayyer, K., Lan, T.-Y., Elser, V. & Loh, N. D. Dragonfly: an implementation of the expand-maximize-compress algorithm for single-particle imaging. *J. Appl. Crystallogr.* **49**, 1320–1335. 10.1107/S1600576716008165 (2016). 27504078 10.1107/S1600576716008165 PMC4970497

[46] Loh, N.-T.D. & Elser, V. Reconstruction algorithm for single-particle diffraction imaging experiments. *Phys. Rev. E* **80**, 026705. 10.1103/PhysRevE.80.026705 (2009). 10.1103/PhysRevE.80.026705 19792279

[47] Hau-Riege, S. P., London, R. A., Huldt, G. & Chapman, H. N. Pulse requirements for x-ray diffraction imaging of single biological molecules. *Phys. Rev. E* **71**, 061919. 10.1103/PhysRevE.71.061919 (2005). 10.1103/PhysRevE.71.061919 16089777

[48] Maia, F. R. N. C., Ekeberg, T., van der Spoel, D. & Hajdu, J. Hawk: the image reconstruction package for coherent X-ray diffractive imaging. *J. Appl. Crystallogr.* **43**, 1535–1539. 10.1107/S0021889810036083 (2010).

[49] Chapman, H. N. et al. High-resolution ab initio three-dimensional x-ray diffraction microscopy. *J. Opt. Soc. Am. A* **23**, 1179–1200. 10.1364/JOSAA.23.001179 (2006). 10.1364/josaa.23.001179 16642197

[50] Liao, H. Y. & Frank, J. Definition and estimation of resolution in single-particle reconstructions. *Structure* **18**, 768–775. 10.1016/j.str.2010.05.008 (2010). 20637413 10.1016/j.str.2010.05.008 PMC2923553

[51] van Heel, M. & Schatz, M. Fourier shell correlation threshold criteria. *J. Struct. Biol.* **151**, 250–262. 10.1016/j.jsb.2005.05.009 (2005). 16125414 10.1016/j.jsb.2005.05.009

[52] Hantke, M. F., Ekeberg, T. & Maia, F. R. N. C. Condor: a simulation tool for flash X-ray imaging. *J. Appl. Crystallogr.* **49**, 1356–1362. 10.1107/S1600576716009213 (2016). 27504081 10.1107/S1600576716009213 PMC4970500

[53] Kuster, M. et al. The 1-Megapixel pnCCD detector for the Small Quantum Systems Instrument at the European XFEL: system and operation aspects. *J. Synchrotron Radiat.* **28**, 576–587. 10.1107/S1600577520015659 (2021). 33650570 10.1107/S1600577520015659 PMC7941295

[54] Allahgholi, A. et al. Megapixels @ Megahertz – the AGIPD high-speed cameras for the European XFEL. *Nucl. Instrum. Methods Phys. Res., Sect. A* **942**, 162324. 10.1016/j.nima.2019.06.065 (2019).

[55] *European XFEL*. EXtra-geom. https://extra-geom.readthedocs.io/en/latest/.

[56] Ho, P. et al. The role of transient resonances for ultra-fast imaging of single sucrose nanoclusters. *Nat. Commun.* **11**, 167. 10.1038/s41467-019-13905-9 (2020). 31919346 10.1038/s41467-019-13905-9 PMC6952381

[57] Mall, A. *et al.* Observation of aerosolization-induced morphological changes in viral capsids (2024). arxiv:2407.11687.

[58] Bean, R. J., Aquila, A., Samoylova, L. & Mancuso, A. P. Design of the mirror optical systems for coherent diffractive imaging at the SPB/SFX instrument of the european XFEL. *J. Opt.* **18**, 074011. 10.1088/2040-8978/18/7/074011 (2016).

[59] Luke, D. R. Relaxed averaged alternating reflections for diffraction imaging. *Inverse Prob.* **21**, 37. 10.1088/0266-5611/21/1/004 (2004).

[60] Fienup, J. R. Phase retrieval algorithms: a comparison. *Appl. Opt.* **21**, 2758–2769. 10.1364/AO.21.002758 (1982). 20396114 10.1364/AO.21.002758

[61] Marchesini, S. et al. X-ray image reconstruction from a diffraction pattern alone. *Phys. Rev. B* **68**, 140101. 10.1103/PhysRevB.68.140101 (2003).

[62] Meng, E. C. et al. UCSF ChimeraX: Tools for structure building and analysis. *Protein Sci.* **32**, e4792. 10.1002/pro.4792 (2023). 37774136 10.1002/pro.4792 PMC10588335

[63] Mazza, T. et al. The beam transport system for the small quantum systems instrument at the european XFEL: optical layout and first commissioning results. *J. Synchrotron Radiat.* **30**, 457–467. 10.1107/S1600577522012085 (2023). 36891860 10.1107/S1600577522012085 PMC10000793

[64] Yan, J. et al. Terawatt-attosecond hard x-ray free-electron laser at high repetition rate. *Nat. Photonics* **18**, 1293–1298. 10.1038/s41566-024-01566-0 (2024).

[65] Kuschel, S. et al. Non-linear enhancement of ultrafast X-ray diffraction through transient resonances. *Nat. Commun.* **16**, 847. 10.1038/s41467-025-56046-y (2025). 39833149 10.1038/s41467-025-56046-y PMC11747624

