# Supplementary material for: Impact of gas background on XFEL single-particle imaging
Source: Sci Rep. 2025 Aug 12;15:29559. doi: 10.1038/s41598-025-15092-8 (PMC12343978; doi:10.1038/s41598-025-15092-8)
Supplement: Supplementary file 1 — Supplementary Information. [file 41598_2025_15092_MOESM1_ESM.pdf]

Supplementary Information
for
Impact of gas background on XFEL single-particle imaging
by
T. You, J. Bielecki and F. R. N. C. Maia

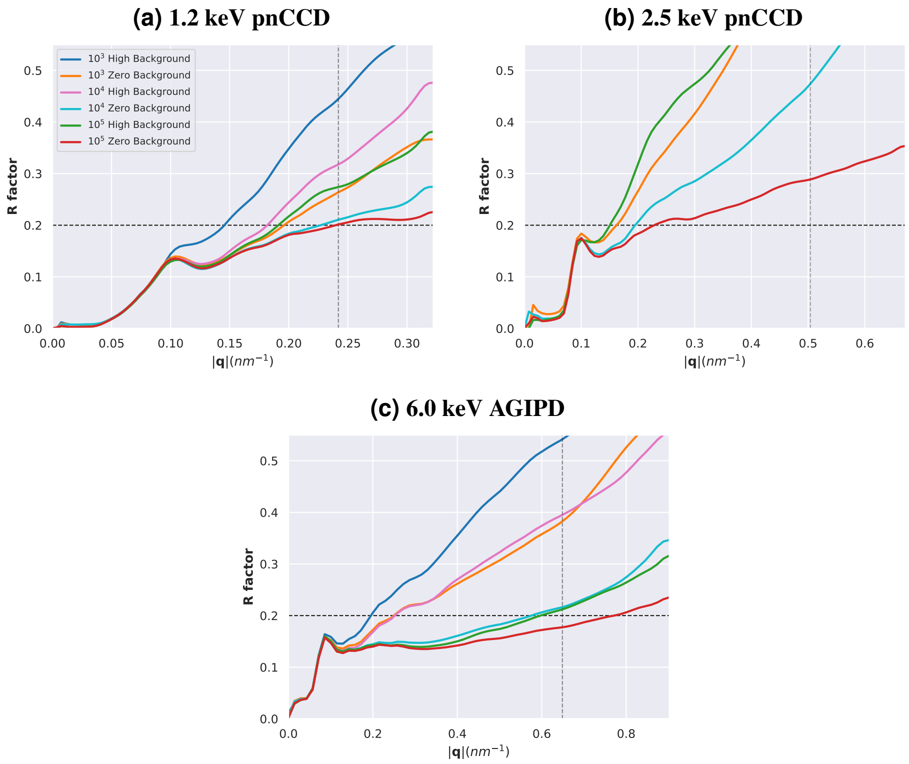

Figure S1. R factor curves for all three energies under high and zero background conditions.

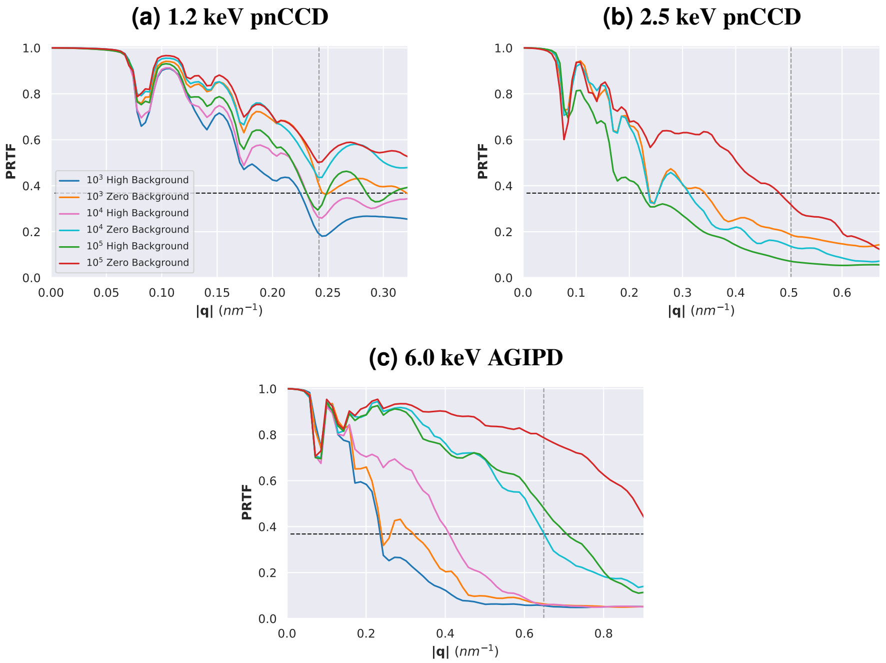

Figure S2. PRTF curves for all three energies under high and zero background conditions.

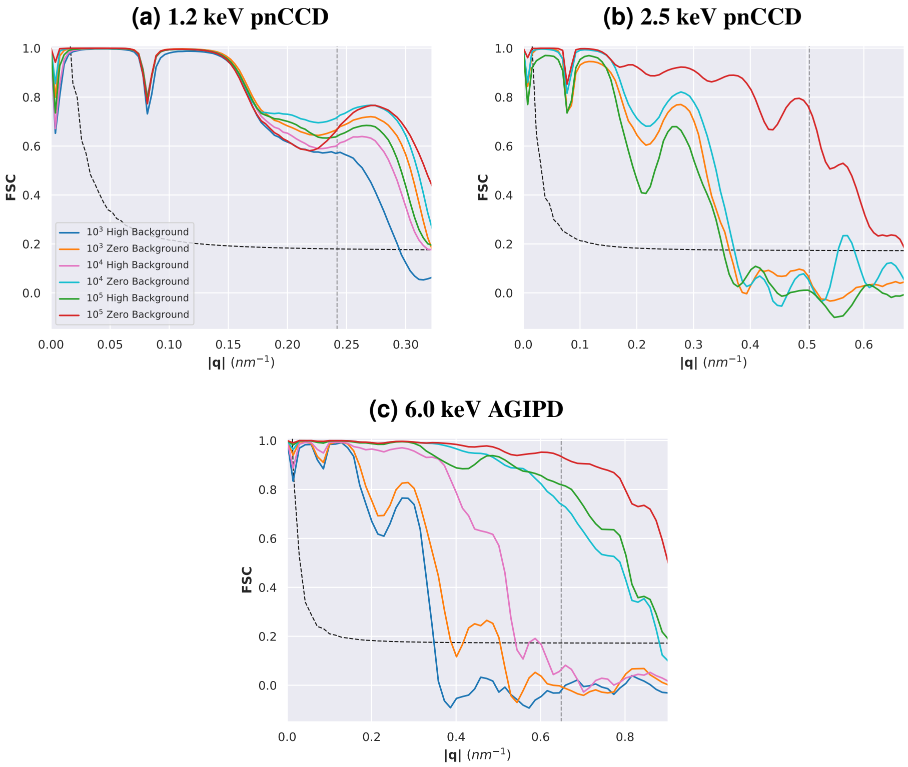

Figure S3. Radially averaged FSC curves for high and zero background reconstructions for all three different energies. The half-bit curve is shown as a black dashed line.

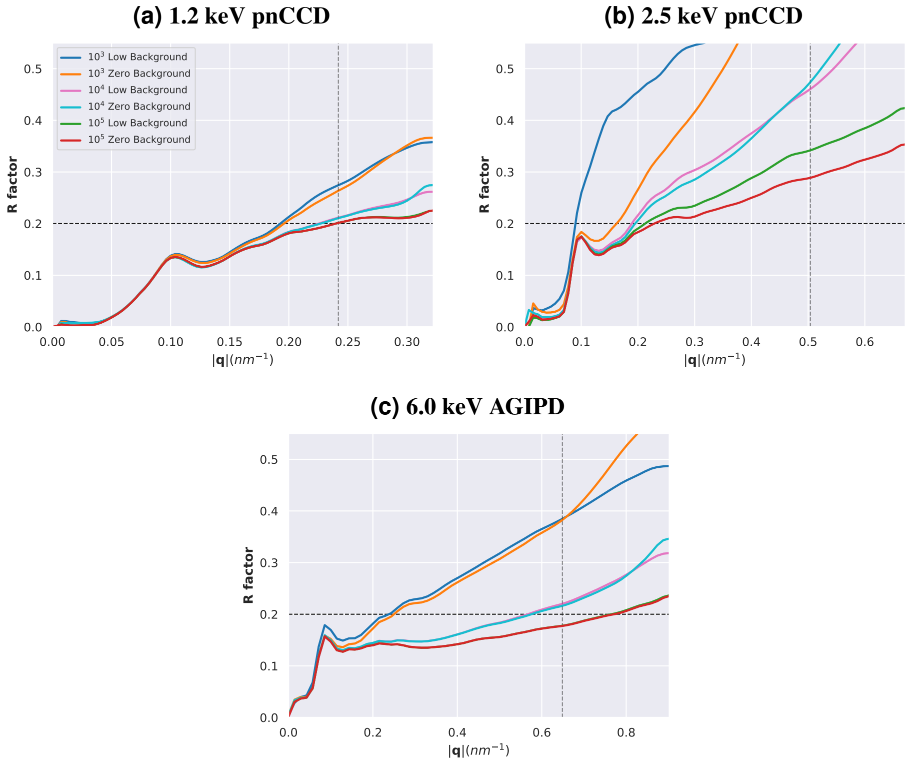

Figure S4. R factor curves for all energies under low and zero background conditions.

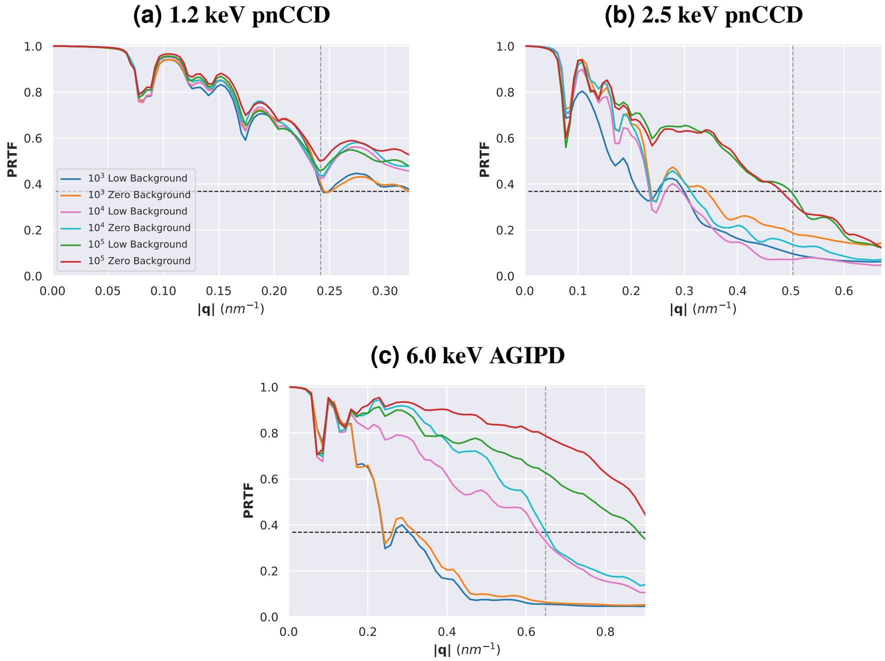

Figure S5. PRTF curves for all energies under low and zero background.

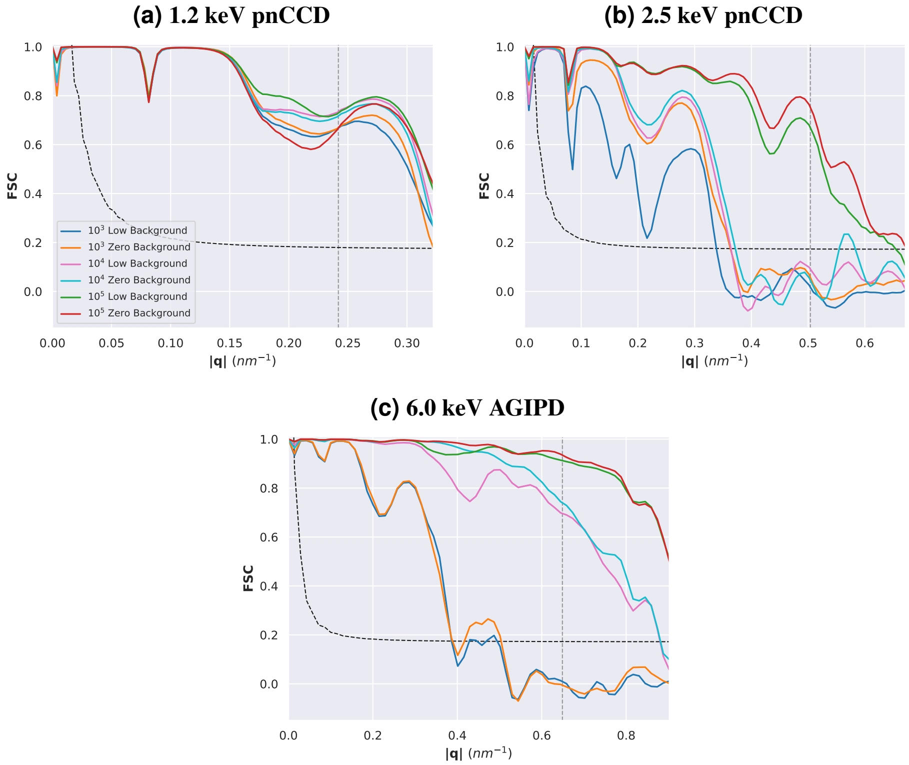

Figure S6. Radially averaged FSC curves for low and zero background for all energies. The half-bit curve is shown as a black dashed line.

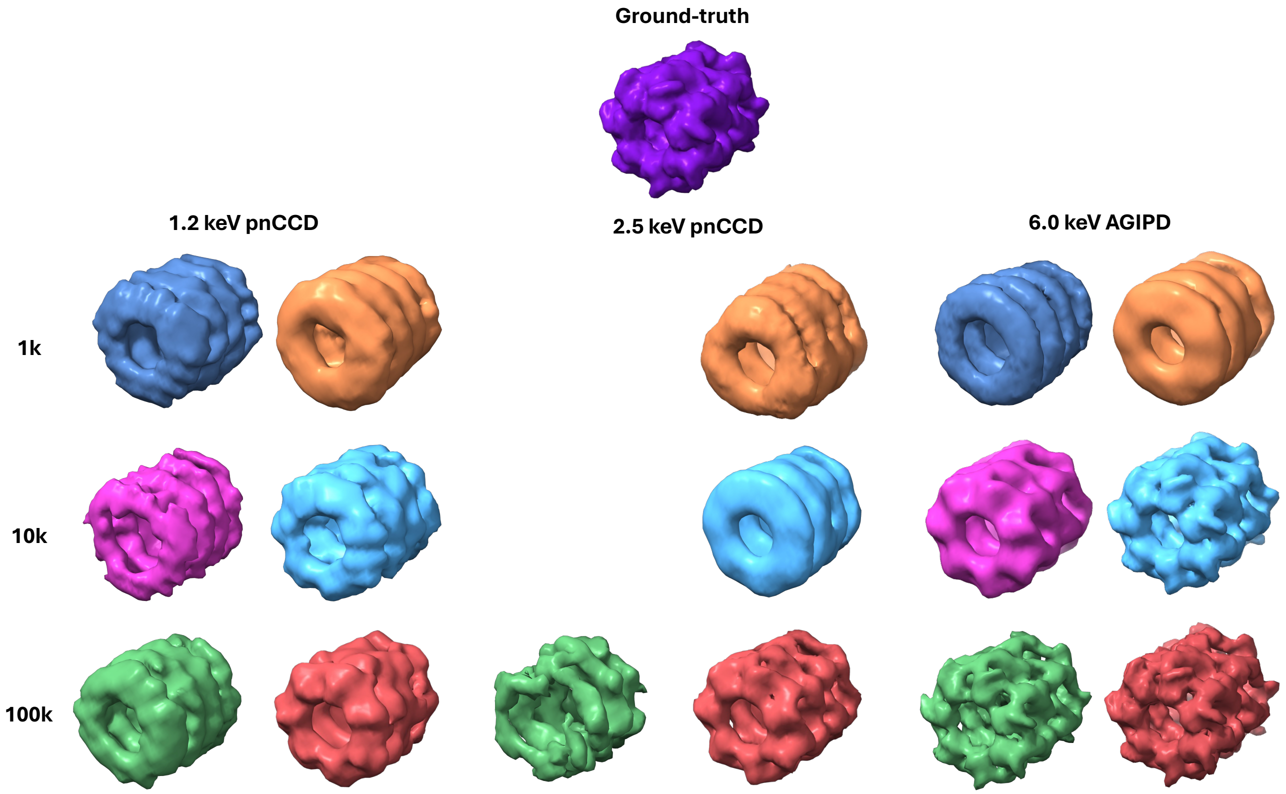

Figure S7. Reconstructed electron density for all three geometries for 10^3, 10^4, and 10^5 pattern reconstructions under high (in blue, magenta, and green on the left) and zero background conditions (in orange, cyan, and red on the right). The ground truth is shown on top of the reconstructed electron densities. The high background reconstructions at 2.5 keV with 10^3 and 10^4 patterns failed to converge and are not shown.

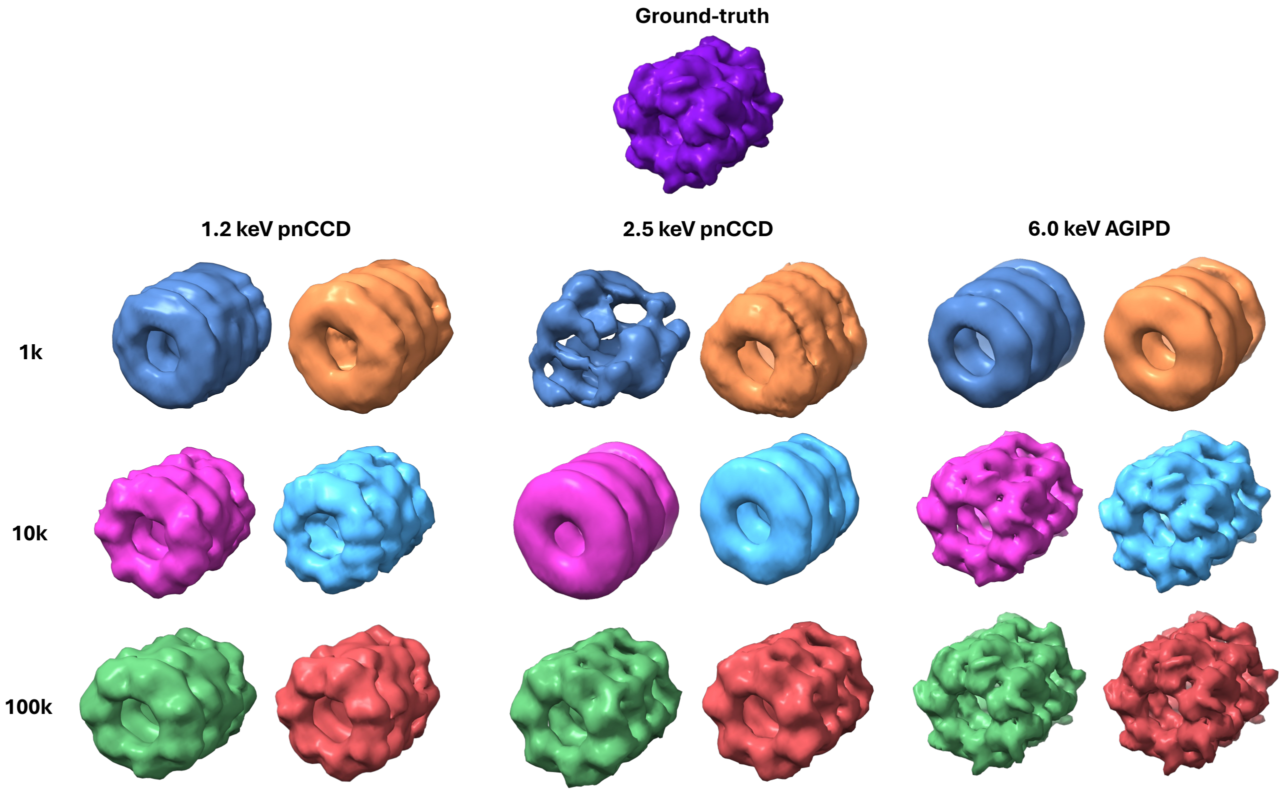

Figure S8. Reconstructed electron density for all three geometries for 10^3, 10^4, and 10^5 pattern reconstructions under low (in blue, magenta, and green on the left) and zero background conditions (in orange, cyan, and red on the right). The ground truth is shown on top of the reconstructed electron densities.

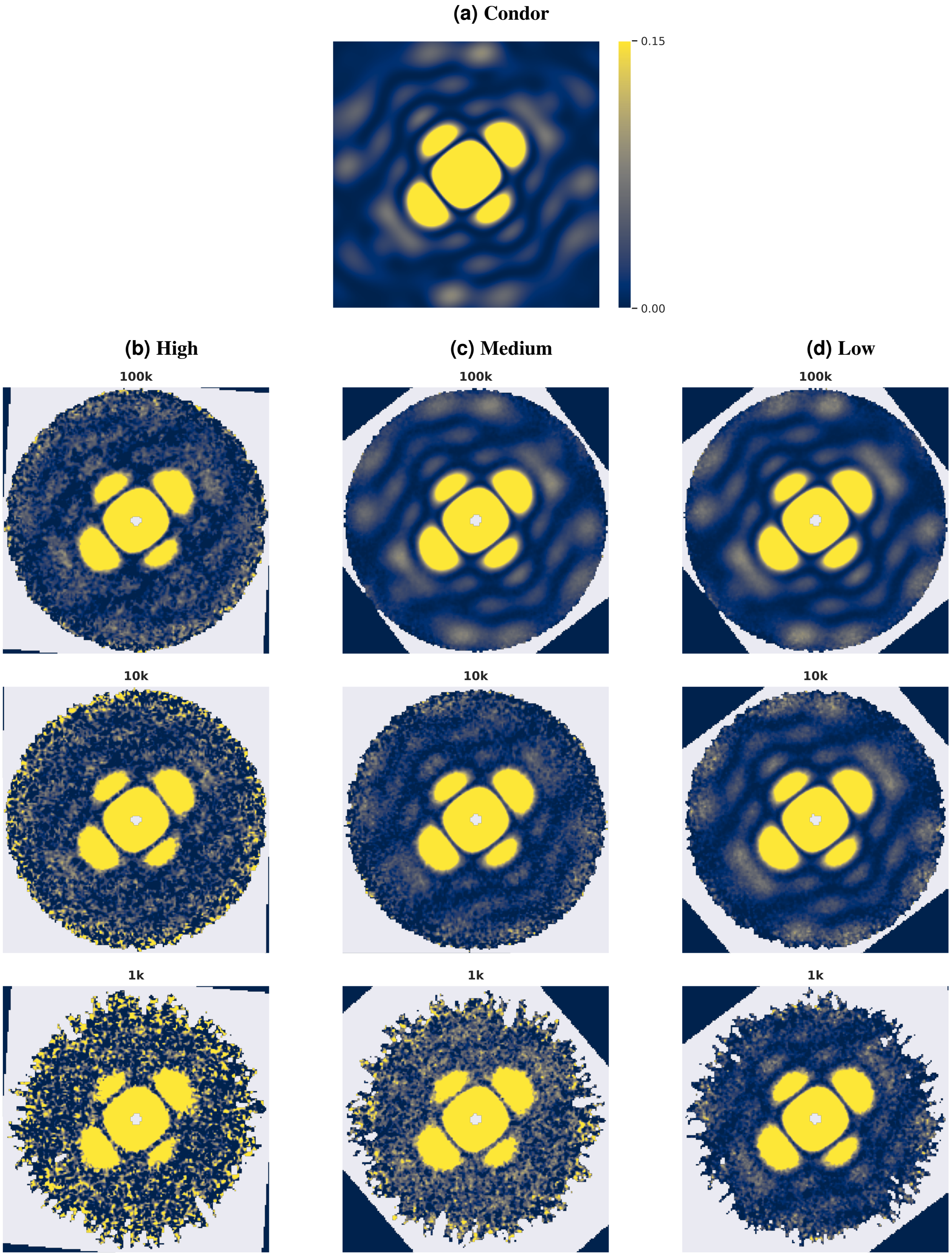

Figure S9. Central XY slices of the Condor ground truth and aligned EMC reconstructions for each background level for 1.2 keV reconstructions. The color map representing intensity has arbitrary units. Each plot has the same minimum and maximum intensity range.

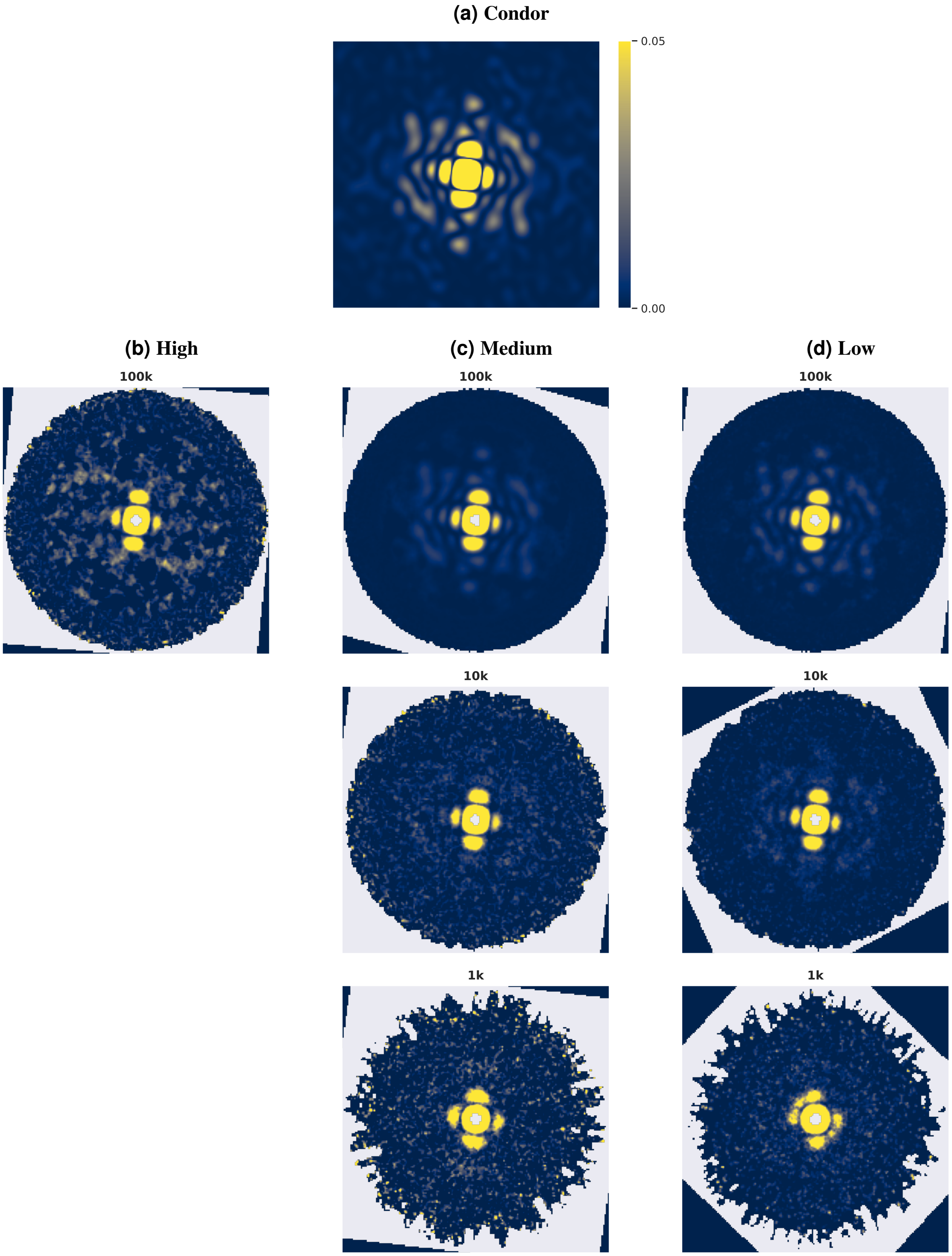

Figure S10. Central XY slices of the Condor ground truth and aligned EMC reconstructions for each background level for 2.5 keV reconstructions. Missing reconstructions are represented by a blank spot in the figure. The color map representing intensity has arbitrary units. Each plot has the same minimum and maximum intensity range.

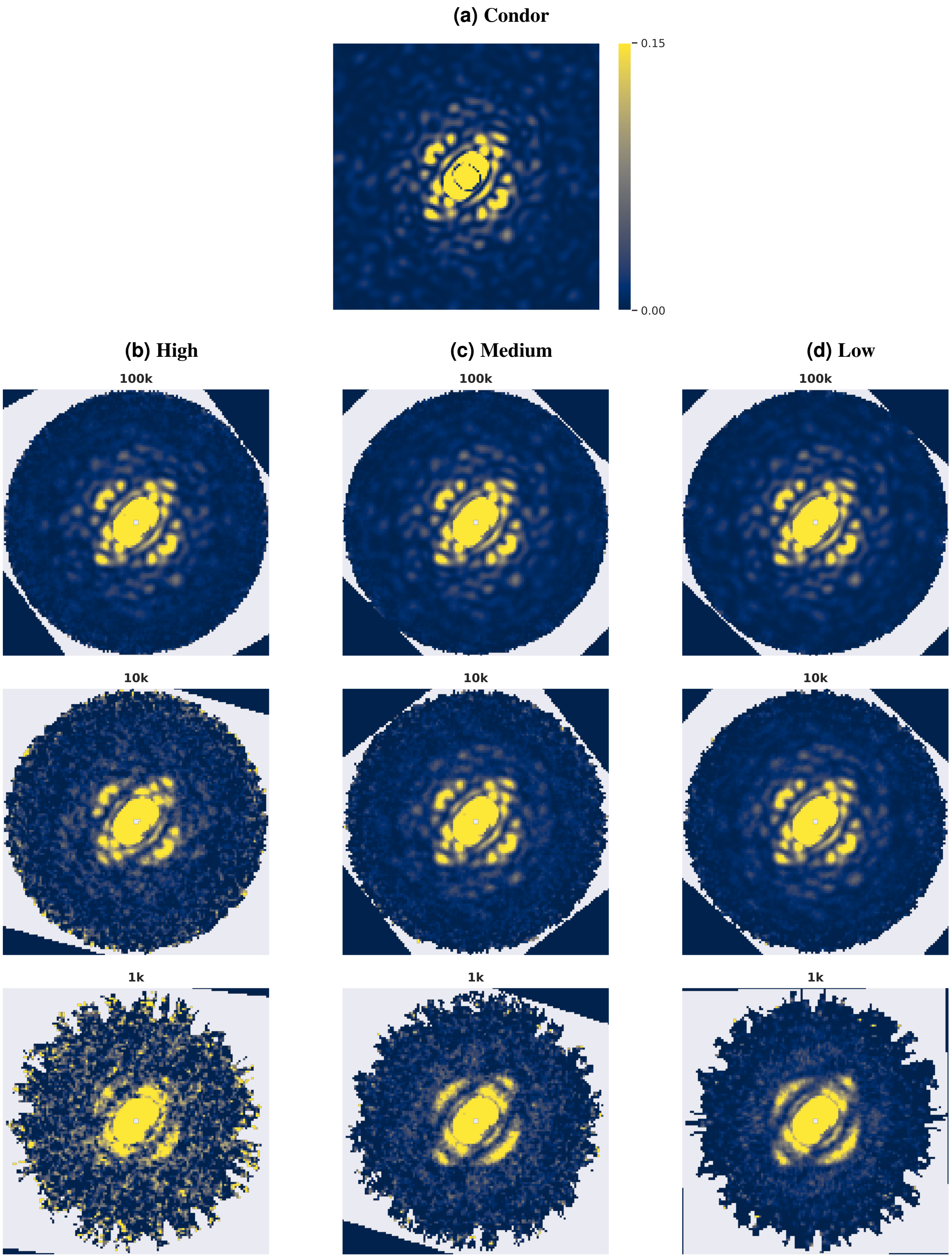

Figure S11. Central XY slices of the Condor ground truth and aligned EMC reconstructions for each background level for 6.0 keV reconstructions. The color map representing intensity has arbitrary units. Each plot has the same minimum and maximum intensity range.
